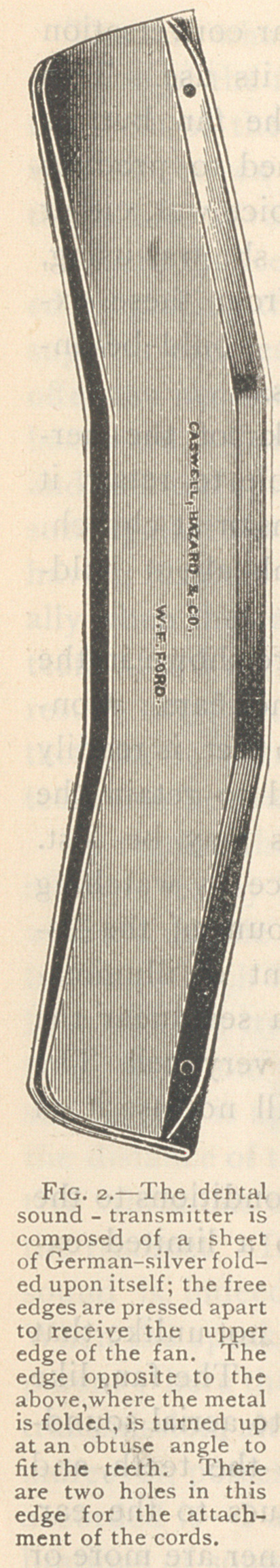# Selections

**Published:** 1881-11

**Authors:** 


### SELECTIONS.

### THE INFLUENCE OF RACE UPON THE ACTION OF POISONS.

The researches of M. Chauveau upon the relative immunity of Algerian sheep from malignant pustule give support to this question. M. Bordier finds that the edible frog (*rana esculenta*) and the ordinary frog (*rana temporaria*) act very differently under the influence of the same quantity of caffein ; while the tree frog (*rana viridis*) is less sensitive to the action of veratrin than the two preceding forms. In Tarentin (according to Darwin) the inhabitants only breed the black sheep because the *hypericum crispum* which abounds there kills off all the white sheep within fifteen days. In Virginia the *lacnanthes tinctoria* kills the white fowls, while black ones eat it with impunity. Cl. Bernard showed that different races of dogs and horses possess distinctive physiological characteristics which were proportional to differences in the properties of certain histological elements, more especially in the nervous system. Negroes can take enormous doses of tartar emetic, and according to Dr. Thaly, they can ingest one gram (fifteen grains) in the course of twenty-four hours with no more effect than would be produced by five centigrams (three-fourths of a grain) in a white man ; they also bear mercury well. Broca also noticed that decomposition sets in more slowly in the bodies of persons belonging to this race. The negro can similarly carry a large quantity of alcohol without being overpowered by it ; and in the black, white, and yellow races an equal quantity of alcohol will not produce a similar state of intoxication. The yellow races can take large doses of drastic medicines.--*Jour. Therap.*

### DENTAL SOUND-TRANSMISSION—THE JAPANESE OTACOUSTIC FAN.*

BY SAMUEL SEXTON, M. D.
Aural Surgeon to the New York Eye and Ear Infirmary.

Mr. President and Gentlemen: The increasing interest manifested in physiological acoustics by otologists during the past few years, has very much quickened the hopes of those who are in a measure cut off from the full benefits of life by impaired hearing; but, although the needs of the deaf have served to stimulate exertions in

* A paper read before the Medical Society of the County of New York, June 26, 1881.

their behalf, the results attained have not as yet fully realized expectations.

For a long time the ear-trumpet, which simply increases the intensity of sound reaching the ear in the natural way, afforded the only practical means of aiding the defective sense of hearing; but this method has never been received with favor, most persons preferring to conceal their defects by avoiding the conspicuousness attending the use of a trumpet. The conversation-tube, which has been long employed, is of very considerable service for private use; but the deaf find that a great many object to placing their mouth to the aperture of a tube in general use.

Of late, however, it has been found that the teeth afford a valuable medium through which the sonorous vibrations of which sound is composed may be transmitted to the auditory nerve. It has been known for a long time that musical tones may be heard by the deaf through the medium of a wooden rod connecting a musical instrument with the teeth; but such means were not found to be available for conversation. The dental transmission of speech by means of a vibrating fan has been frequently observed quite accidentally by persons who have placed the edge of a fan to the teeth while others were conversing; but it remained for Mr. Rhoades to make use of this fact in a practical way, by introducing a fan for conversational purposes. My earlier experiences with the audiphone were not encouraging, for it seemed that but few were willing to adopt them on account of their expensiveness, the advantages attending their use being apparently so limited. But now our Japanese friends, who are not slow in adopting useful discoveries from any source, have taken advantage of their great facilities for the production of all kinds of fans, to manufacture a sound-transmitting fan, the low price of which promises to bring it within the reach of all.

The first Japanese product came appropriately enough from the Lakujenqui Society's Institution for the Education of the Deaf and Dumb. It may be of interest, in this connection, to state that this society was organized some three years ago with Mr. Yamao, the Minister of Public Works, as President. Mr. Maye-Shimea, the Postmaster-General, is an active member of the Board of Trustees, as is also Mr. Yegawa Kimihira, the Japanese Consul to New York City, who is present this evening. The deaf and dumb institution founded by this society in Tokio three years ago, was the first of its kind to be established in Eastern Asia. It is free to the deaf and dumb from all parts of the empire, and is supported by the gifts of the founders and by voluntary contributions—their majesties, the Emperor and Empress, having given it their support. It is conducted entirely by the natives; the sign-language has only been taught as yet, although improvements in teaching will doubtless be made in the future.

It is the intention to make this institution self-supporting by the labor of its inmates, and I have the pleasure of showing you to-night the first products of their skill, which have recently been sent to this country by the society's president.

These sound-transmitting fans were placed in my hands soon after their arrival here, a few months ago, by Mr. Kimihira, that their merits might be tested, the trustees desiring to know if their construction is the best that our present knowledge of the subject can suggest, and, also, whether there is likely to be a demand for them in this country.

Having given these fans a careful trial, and finding that some of them—for there are several varieties—are more satisfactory than any sound-transmitting fan yet produced, and deeming them worthy of a further trial by the profession, I have presented them to the society, with some remarks on their use. I may here state that these fans are made of lacquered material; that they have several shapes, and are of different qualities, but not altogether unlike the audiphone, after which they were modelled. The lacquered sheets of which they are made, seem to answer the purpose even better than the vulcanite of which the audiphone is constructed; they are by far the more durable of the two, and much cheaper—the lowest in price being the best. They range in price from thirty-three cents up to three dollars each.

The method of using all sound-transmitting fans is similar; the fan is first bent upon itself so that its surface shall form a curve a little greater than the arc of an ellipse, but not quite equal to the arc of a circle, in which position it must be securely fixed by attaching the cords coming from the superior border to a peg on the handle. The curvature should be unchanged while the fan is in use, else the person employing it, not feeling at ease, will fail to obtain its full advantages. During a protracted use of the fan the arm should have a rest, experience showing that those who require its aid are liable to become very nervous, and should not, therefore, have to think about keeping the fan in its place, etc.

FIG. 1.—Lateral view of the fan when held in position for dental sound transmission. The dental sound-transmitter, which is attached to the fan in the cut, is somewhat exaggerated in size, and its proper position between the canine and lateral incisor teeth of the upper jaw is not well shown. E. Location of entrance to external auditory meatus.

The fan, when bent as above described, is ready for use. The position of the fan, when its superior border has been placed against the teeth of the upper jaw, is very important. It should be held with its convex surface presenting to the speaker, who will be heard to the best advantage when directing his voice toward it.

Where the teeth are in good condition, the best results are obtained by placing the edge of the fan against either one, or both, of the superior canines, which are a little longer than the incisors, and thus offer the necessary resistance when the fan is pressed upward and backward. Contact with the teeth should be maintained by gentle and uninterrupted pressure while the fan is in use. Too much pressure against the teeth is to be avoided, a neglect of this precaution having resulted in loosening or otherwise injuring the teeth, especially when they are not in a healthy condition. Although the canine teeth afford the best medium for dental sound transmission, good results may be obtained from any of the upper teeth with which contact can be obtained. The teeth of the lower jaw and artificial teeth can be availed of to a limited extent only.

As regards the actual range of usefulness of dental sound-transmission, it may be said that its best results are obtained with those who are unable to hear the voice when shouted, say at a distance of ten feet ; such individuals can usually hear words at that distance best with the trumpet, but when the speaker is removed only two or three feet from them, the fan affords the most assistance. The reason for this is apparent when the conditions are considered ; for at the distance of ten feet the sound-waves are too feeble to cause effective vibrations of the fan, but they are collected and intensified by the trumpet. In either case the results are not very satisfactory : sound is, indeed, plainly heard, but definition, so to speak, is lost. When the speaker is near, however, the advantages of the fan are apparent ; here the trumpet has a disagreeable resonance, while dental transmission, although imperfect, is more natural. The latter method, moreover, possesses an advantage that has not, to my knowledge, been accorded it—I allude to the bin-aural character of its action, the vibrations of sound passing to both ears at the same time.

Some individuals seem to be unable to acquire the use of either the fan or the trumpet. In one instance which I recall, the patient, a lady, could only hear conversation near by when the voice was raised to a loud tone ; with the trumpet she could hear conversation when low, but its cumbrousness was an objection to its use. This lady heard ordinary conversation very plainly with the fan, but its metallic timbre was disagreeable, and the voice seemed to produce reverberations in her head ; the more distinct my voice was, either by raising its pitch or by a nearer approach to the fan she was using, the more disagreeable it became. She concluded, from these experiments, that hearing in this disagreeable manner would be insupportable, and that it would make her very nervous.

Another patient, who uses the fan constantly, speaks of the nervous symptoms that arise from its use when he is unable to retain it in its proper position with ease, as in long conversations or at church. He can hear much better when not compelled to think about holdit in its proper position.

The fan's advantages, even in very great deafness, are shown in the case of a lady who can only hear words shouted into her ears. Conversation with the fan, however, at the distance of two feet, is readily carried on ; at a greater distance, even, she is enabled to retain the thread of conversation, although some of the words may be lost. Like all very deaf persons, she gains much assistance by watching the speaker's lips, and when unable to do this, on account of the distance of the speaker—she is near-sighted—or the want of illumination, the fan is of much less service. By occupying a seat near the pulpit she has the satisfaction of hearing the minister very well. The trumpet aids her rather more than the fan, but she will not use it on any account.

Persons with normal hearing possess the requisite conditions to the perception of sound transmitted through the teeth to a limited extent only.

The *modus operandi* of dental sound-transmission is not unlike that of the normal ear, but it is far from being so delicate. The fan, like the membrana tympani, moves to and fro in response to aerial sound-waves, transmitting the impressions thus received to the teeth, and thence mainly through the intervening osseous tissues to the ear. The sounds that reach the auditory nerve in this manner are more or less modified as compared to normal transmission ; the timbre is sometimes described as "metallic." Musical tones, however, seem to be most plainly heard.

FIG. 2.—The dental sound - transmitter is composed of a sheet of German-silver folded upon itself; the free edges are pressed apart to receive the upper edge of the fan. The edge opposite to the above, where the metal is folded, is turned up at an obtuse angle to fit the teeth. There are two holes in this edge for the attachment of the cords.

Osseous transmission is insufficient for the more delicate shades of speech ; thus hissing sounds are generally suppressed. But notwithstanding its imperfections, the aid it offers to the deaf is of much service, and I have found many individuals who, by the use of a fan, enjoy a degree of hearing not otherwise obtainable. In these instances, however, the successful use of the fan has been only acquired by the observance of the laws which govern sound-transmission ; such a course enables the patient to ascertain just what benefit may reasonably be expected from the method, and to thus avoid the nervousness and disappointment which so often arise from futile attempts to obtain results in their nature impracticable.

These conclusions are somewhat at variance with the opinions I formerly entertained on this subject, but my later experience seems to justify them,

The experiments that I have recently made with Japanese and other fans, have led me to devise a dental sound-transmitter which can be attached to almost any fan. It is so constructed that it may be readily slipped over the upper edge of the ordinary paper-covered bamboo fan, when, by means of the cords fastened to it, the fan may be bent over and secured ready for use. When not in use it can be carried in the pocket. The springiness of the two plates of the instrument is sufficient to retain it securely in its place on the fan. It consists of one piece of German-silver plate folded together, but leaving space enough to receive the edge of the fan between its free edges. The mouth-piece of the sound-transmitter is slightly turned up, which affords a more perfect adaptation to the teeth than the edge of a fan ; it also protects the fan from moisture and from injury by the teeth. It is made by Mr. Ford, of Messrs. Caswell, Hazard & Co. It is nickel-plated.

By means of this instrument a great variety of fans become available for the transmission of sound; I have found the ordinary Japanese fan of commerce, which costs but five cents, to answer as well as any other, although not so durable as the Japanese otacoustic fan, which is the best for the use of gentlemen who wish to carry the fan about with them in a special pocket made on the inside of the skirt of the coat. A fan of medium size has been found more convenient than the larger ones.

*Conclusions.*—Dental transmission of sound, for conversational purposes, is of very considerable advantage to a large number of very deaf individuals. Its inconspicuousness gives it a decided advantage over the trumpet.

In most instances it does not convey as great a volume of sound to the ear as the trumpet, but, notwithstanding this fact, most patients prefer the fan. By either method general conversation cannot be well heard, the patient being compelled to confine his attention to one speaker at a time.

In adopting dental transmission as an aid to the impaired sense of hearing, the assistance of some one competent to give instructions respecting the best method of using the fan should be obtained.

And finally, only the limited amount of assistance should be promised which experience has shown to be practicable.—*Medical Record.*

### CURIOUS SANITARY DISCOVERIES.

A municipal laboratory was not long since opened to the public in Paris, and many of its discoveries have been as curious and unexpected as they promise to be useful. For a small fee anybody can have analyzed samples of food, drinks, or anything connected with public hygiene. Among the articles in which adulteration was chiefly detected were wine, butter and milk. The adulteration of wine consists principally of its being colored with fuchsine, and in many cases it does not contain a particle of the juice of the grape. Milk is deprived of its cream, and abundantly watered, and butter is frequently composed of anything but the ingredients of milk. Several samples of butter were found to be made with oil or suet, which were easily separated, and shown to the visitors, who accompanied the Prefect of Police to witness the experiments that were being performed in the laboratory. A sample that was exhibited as cream, which appeared as natural as possible, and of excellent flavor, was declared to have been manufactured with the residue of some red dye, mixed up with oil and sulphuric acid, in certain proportions. Some children's toys were examined, and those painted with red and blue were almost always found to contain poisonous substances, consisting principally of lead and copper. Even articles of perfumery were examined, which brought to light the most extraordinary examples of the skill brought into requisition for the accomplishment of fraudulent practices, for in several samples of scents of the most exquisite perfume, they were found to be manufactured with other materials than the essential oils of the flowers which they were intended to represent. But the most important discovery made was the presence in the nipples of some feeding bottles of a mass of vegetation, of cryptogamic nature, which, according to Dr. Faurel, bears a great analogy to the aphthous condition of the mouth frequently found in infancy, and he has induced the Academy of Medicine to investigate the matter, as he believes the condition referred to to be the origin of the intestinal affections, and particularly that form called "athrepsia," to which infants brought up by the bottle are subject.—*Med. and Surg. Rep.*

### RUPTURE OF THE UTERUS TREATED BY DRAINAGE.

On March 26th, A. C——, a woman at full term, was brought into C. von Hecker's wards, in Munich, in a state of extreme collapse, with the objective signs of rupture of the uterus. The lower extremities of the fœtus could be felt immediately behind the abdominal parietes in the right hypochondriac region. On the left side the firmly contracted body of the uterus was felt, and just above it a soft mass was perceptible, evidently the placenta. Per vaginam, the fetal head was found just above the inferior strait. It was stated that previous to the arrival of the patient in the hospital the pains had been uncommonly strong, and had been further stimulated by the administration of ergot At the time of admission, however, the uterine contractions had ceased, as is usually the case when rupture has taken place. The forceps was immediately applied, and three tractions sufficed to deliver the head, the rest of the body following quickly. Vaginal examination now disclosed a large rupture in the cervix, penetrating through all the coats; it was extensive enough to have admitted the passage of the hand into the peritoneal cavity. The fingers were introduced through the rent, following the umbilical cord to the placenta, which was situated on the left side, as previously diagnosed, and was readily extracted. The condition of the patient was not materially altered by these manipulations, so that stimulation was not resorted to. Next morning the abdomen became very painful and tympanitic, and vomiting of green fluid, which had been present previous to delivery, recurred. Accidental circumstances prevented the introduction of a drainage tube until thirteen hours after delivery. When once in place, however, it gave exit to a considerable quantity of sanguinolent fluid, which continued to drain away slowly for some time. There were frequent calls to empty the bladder, so that the catheter was finally resorted to and enormous quantities of urine evacuated. During the next few days iced compresses and opium were sufficient to overcome the peritoneal irritation. There were profuse and fetid fecal evacuations, which seemed to exercise a favorable influence on the condition of the patient, but which caused the extrusion of the drainage tube on March 30th. On reintroduction of the latter more sanguinolent fluid escaped, but the quantity rapidly decreased. The temperature did not rise above 38 deg. C., and in two weeks the rupture was firmly cicatrized. The above case shows that drainage is the cardinal point in the treatment of rupture of the uterus, as the danger in these cases consists in the retention of septic fluids in the peritoneal cavity. Of late several successful cases have been reported in which the line of treatment pursued was essentially the same. In some of them the abdominal cavity was also washed out with antiseptic lotions, usually two per cent. solutions of carbolic acid. Among the advantages of this method of treatment are its simplicity and easy applicability, which recommend it to the country practitioner in whose practice such cases are especially apt to occur, because of the long time which often necessarily elapses before the services of a physician can be secured. If possible, however, irrigations of the peritoneal cavity with carbolized solutions should be dispensed with, as they are by no means free from danger.—*Centralblatt fur Gynakologie*, May 14, 1881.

### AMPUTATION AT THE SHOULDER-JOINT FOR CANCER OF THE AXILLA.

Dr Stephen Smith reports (*Med. Gazette*) two cases of recurrent cancer of the axilla, in one of which amputation at the shoulder-joint was performed with careful removal of all infiltration about the axilla. The patient has not had a recurrence in nine years, although the growth had previously been removed by dissection five times. In the other case the same operation was urged but refused.

### RECTAL EXPLORATION AND DIAGNOSIS.

Dr. Charles B. Kelsey, of New York, contributes an article to the "*New York Medical Journal and Obstetrical Review*" for October, 1881, in which he attempts to answer the question of how to make a rectal examination which shall be at the same time thorough and as free from pain as possible. In his own practice he uses an artificial light of his own arrangement and a forehead mirror, which enable him at all times to illuminate the rectum thoroughly, while by the side of the examining table stands an instrument case fitted with all necessary appliances. In addition to these things he insists strongly on the necessity of having a water-closet communicating with the office, so that injections may be administered and the bowels moved at the time of the examination. In the matter of specula he confines himself almost exclusively to Sims', finding this the best of all after the sphincter has been stretched, and not finding any that give a fair view of the parts until this has been done. He relies, however, much more upon the finger for a diagnosis than upon any artificial helps, and claims that with it, after the necessary skill has been acquired, the slightest pathological changes may be detected. In the matter of bougies he also has his own preference, and recommends a soft-rubber instrument, similar to that of Wales, only more flexible. For detecting strictures high up in the rectum or in the sigmoid flexure little confidence is to be placed in a bougie of any sort, and the writer relies almost entirely upon manual examination, either through the abdominal wall or by passing the hand into the rectal pouch. The latter method he holds to be free from danger and certain in its conclusions.

### HOW SOON AFTER DELIVERY SHOULD A WOMAN GET UP.

We know that the majority of chronic affections of the genital organs from which the females of all classes so often suffer, coincide in their beginning with the epoque of one of their lyings in, and nothing, other than this circumstance, would have obliged me to consider, so seriously, the question of what treatment a lying in female should be subjected to ; more especially as the knowledge of the etiological epoque of any disease, will enable the same to be guarded against by due care ; it must, however, be admitted that physicians cannot always solve this question in a satisfactory manner. We often content ourselves with attributing the origin of a disease to certain facts that explain it in a more or less satisfactory manner as a puerperal disease, or some operation performed during the lying in, premature coitus, etc.

But in the case of an operation performed during the lying in, to determine if it be the cause of the disease, is not always easy. How, for example, can it be determined whether a vesico-vaginal fistula has been occasioned by the use of forceps, or by the pressure on the bladder of the child's head, or why in certain cases the employment of forceps is followed by a disease of the uterus, when in other cases where forceps have been used in a case of difficulty, in which decapitation has been had recourse to, the health of the woman does not suffer, although the conditions are apparently the same.

In all cases it is certain the early quitting of her couch by the lying in female has been the cause chosen by gynæcologists, physicians, and midwives to explain the appearance of a chronic disease in the female genital organs ; in short, when occupied with the etiology of female diseases when the fact is confirmed by the responses of the female to the questions of the physician, and this is particularly the case when dealing with females living by their labor, it is very easy to attribute the cause of these afflictions to too early rising after delivery. But do not we here encounter a series of facts tending to contradict this ; then if it were absolutely true, all the poorer class of females would require medical treatment ; but I doubt very much whether the greatest number of chronic uterine affections or catarrhal uterine diseases, syphilis excepted, are to be found among this class. Unfortunately exact statistics on this point are wanting ; it can be said, however, that females of the non-laboring class are attacked by these same diseases, although they remain in bed precisely the nine days allotted by usage, even in cases where they are permitted to rise before this time.

It is well known, also, that females of the laboring classes are under the necessity, not only of rising soon after their lying in, but also of engaging in very laborious work, scrubbing floors, washing large quantities of clothes, etc., and to these latter factors I attribute the origin of the at first acute but secondarily chronic affections of the uterus. From these circumstances it seems safe to conclude that it is not to a premature rising that the physician can refer to as causes of uterine disease, but to other circumstances which a strict search is required to determine. As to the question of lying in for nine days, it seems to me that according to certain circumstances which I shall cite later on, it would appear that lying in continued for nine days is not only useless but in some cases it is hurtful to the organism, and that it may even occasion diseases of the uterus and neighboring organs.

For a long time this obligation of remaining so long a time in bed after delivery has seemed to me unjustifiable. Goodell, of Philadelphia, has pronounced a similar opinion, and he allows the female to quit the lying in couch when she desires. According to him a too long prolonged horizontal posture may have disagreeable consequences ; and as the lying in is purely a physiological act, nature shows this fact that the desire of the patient to rise frequently anticipates the permission of the physician. This wish to rise should be regarded as a perfectly sufficient reason for the patient quitting her bed. Goodell's personal experience shows, he claims, that the female is often stronger on the fifth day than the ninth day after delivering, if she has been kept in bed. In defence of his opinion, Goodell cites the fact that the vertical position not only excites uterine involution, but by regulating the circulation it diminishes the quantity of the lochia and shortens the time during which they are evacuated. On the contrary the horizontal position produces a passive hyperæmia of the uterus, a stagnation of the blood in the posterior parieties, which, besides this, is already hyperæmic from the attachment of the placenta ; in a word this position does all that can be done to interfere with a regular progressive puerperal uterine involution. In support of his opinion, Goodell cites the fact that diseases of the uterus are almost unknown in countries where women rise soon after delivery. It seems proven by the classics that among the Greeks and Romans, the women quitted the bed soon after delivery, to plunge in running water. But what is most conclusive of all is the fact ascertained by Goodell, that the health of the patients following the treatment indicated by him was soonest re-established.

Although the opinion of Goodell seems plausible, and probable enough of itself, he supports it by figures. As far as concerns myself, as I have already said, practice has taught me that patients ordinarily charge their diseases to the beginning of their lying in, although they can be directly traced in many instances to a premature and painful labor, in addition to theoretical considerations, which tend to refute the prolonged lying in theory, may be added in my opinion, pathological evidences. Kehrer's experiments on hares with lochial secretions, for example, have led him to the following conclusion : The blood which flows from the genital organs soon after delivery, if injected into the skin of the rabbet, produces at first a slight redness of the skin of the hare, which soon changes into a nodus that disappears at the end of a few days. The lochia which appears during the next days, even in cases where the puerperal condition has been normal, produces extensive inflammation and abscesses of the subcutaneous cellular tissue. The action of the latter lochia does not differ from that of the ichorous and putrid lochia of the earlier period and in proportion to the duration of the lying in period, the lochia become more and more contagious after killing the animals on whom the experiments are made.

These experiments of Kehrer seem sufficient for the end we have proposed for them, even laying aside the question of the nature of the poison contained in them ; in short, if we concluded that the contagious nature of the lochia is proved by experience, and if we consider the naked condition of the mucous membrane of the uterus, the bruised condition of the vaginal mucous membrane, it will be easily understood how puerperal inflammation and fevers are caused by auto-inoculation. Here is the question : In what condition will the uterine lochia flow most freely, in the vertical or horizontal position of the woman ? In my opinion the vertical position facilitates the puerperal evacuation. Thus it has more than once happened under my observation, that when a clot has been retained in the uterus, causing a hæmorrhage, raising the woman to the vertical position, has caused the clot to be expelled and the hæmorrhage to cease.

In general, it may be said that when the patient keeps her bed for nine days, that the uterus is less diminished in size than the uterus of the woman who rises in less than that period, which shows that early rising after delivery, tends to facilitate rather than delay uterine involution. The conclusions are derived from results obtained from two hundred and thirty-five cases. I should say that each woman before leaving the hospital, was submitted to a gynæcological exploration to determine whether any uterine displacement existed, and that in no case could an abnormal position be observed. To add to the value of these facts, I may say that among many of our patients, fever made its appearance the second, third, or fourth day after delivery, which did not yield to quinine or anti-septic lotions; the fever ceased, however, when the patient quitted her bed.

This latter fact indicates that in all probability the fever has its origin in matters harmful to the organism, and seems to prove that the uterine lochia may be the source of these matters, and that the elevation of the temperature may be explained by the absorption of the lochia by the vessels of the abraded mucous membrane. Basing my opinion on all that precedes, I conclude that it is not rational to follow this old error, that is, to force females to keep their bed during nine days, and I therefore allow them to quit their bed whenever they so desire, provided there is nothing to contra-indicate this. In conclusion I may say that every patient soon after delivery is submitted to the use of lotions of permanganate of potash, in order to destroy the hurtful qualities of the lochia as far as possible. It is impossible to indicate the exact period when a woman should quit her bed, but that this should be determined by questions other than time.—*American Medical Bi-Weekly.*

### THE IMMEDIATE CURE OF INGUINAL HERNIA.

Nearly a year ago, Mr. W. Dunnett Spanton, M. R. C. S., reported (*Brit. Med. Journal*, Dec. 11, 1880,) a number of cases of inguinal hernia in which a radical cure was achieved by means of a novel method. The instrument used is shaped somewhat like a corkscrew, with a flat point and movable handle. The screw is rather broader near the point, tapering somewhat toward the handle, and should be sufficiently strong not to break, but yet as thin as may be consistent with strength.

The mode of performing the operation in a case of ordinary oblique inguinal hernia is as follows: The patient must be in good health, have an aperient the day before, and an enema on the morning of operation. If necessary, the pubes must be shaved. Under the influence of an anæsthetic, the hernia is carefully reduced, and not allowed to come down during the operation. An incision is made in the skin of the scrotum large enough to admit the forefinger easily, over the fundus of the hernial sac, generally about two inches below the spine of the os pubis; and the skin is separated from the parts beneath by means of the blade or handle of a narrow scalpel, to an extent determined by the size of the hernia, and that of the inguinal canal. The operator standing on the left-hand side of the patient, the forefinger of the left hand is passed up to the internal abdominal ring, invaginating the fascia and hernial sac to the same extent. A careful examination is now made of the surrounding structures, the position of the vessels clearly made out, the size and shape of the abdominal rings noted, as well as the length of the canal. This is necessary, in order to have an instrument of the proper size. The left forefinger being retained in the hernial canal, protecting the spermatic cord, and at the same time closing the internal ring, the screw instrument, previously dipped in carbolic oil, is, with the right hand, thrust through the skin of the groin so as to transfix the aponeurosis of the external oblique muscle, at a point somewhat above that at which it is intended to pass through the conjoined tendon. Having giving the instrument one half turn to the right, if a right inguinal, and a whole turn if it be a left hernia, it is next made to pierce subcutaneously the conjoined tendon of the internal oblique and transversalis muscles as high up as can safely be reached, the left forefinger carefully guarding the point, so as to avoid wounding the vessels or peritoneum. This part of the operation must be executed cautiously. It will then be found that, as soon as a hold has been secured by the instruments, the internal ring is practically closed. Another turn is now given to the screw, causing it to pass through the invaginated tissue—whether consisting of fascia, or sac, or both—and it is again passed through the external pillar, and then across to the internal pillar of the external ring, and another turn given, if possible, so as to bring the point out at the wound in the scrotum. The handle should then lie flatwise on the abdomen, and the point of the instrument be protected by a round piece of solid India-rubber, or by winding round it some carbolized gauze. A light pad is then placed over the part, and a bandage carefully applied. The amount of induration excited will be the guide as to the time for removal of the instrument; but a week has been usually found sufficient. The removal of the instrument is easily effected, as the suppuration which takes place along its course serves to loosen it somewhat; and by keeping it well oiled from day to day, it is easily withdrawn. The wounds will heal under any simple dressing, with pad and bandage. The aim of the operation is to bring together the pillars of the hernial canal, and at the same time to plug the opening in such a manner as to shut it off from the peritoneal cavity on the one hand, and on the other to form an impassable barrier against any further descent of the bowel. So long as the general peritoneal cavity is not interfered with, so far is danger averted; and, if the hernial canal be effectually closed throughout, so to the like extent is the cure complete.

At the recent meeting of the British Medical Association, Mr. Spanton related nine additional cases in which his operation had been performed, making a total of thirty-four cases. Of these thirty had been quite successful, and the remaining four much benefited. There had been no death. In some of the patients no truss had been of the slightest use. A tendon or catgut ligature was employed in three of the cases, passed in a similar manner to the screw, and retained *in situ* until the parts become consolidated. The result in these cases was found to be on the whole less satisfactory than in those treated by the screw alone. In them, however, the beneficial influence of Listerism was most marked, and the author advised its use in every such operation in which an animal ligature was employed. The paper concluded with an appeal to surgeons to give the operation for radical cure a fair trial, and not to rest satisfied with recommending the mere use of trusses; more especially to urge parents, in the case of young children, to have them cured while young by some operation which had been proved to be both safe and effectual. In the discussion which followed the report of the cases, Mr. Harrison (Liverpool) remarked that thirty out of the thirty-four cases had been spoken of as "more or less" effectually relieved, and inquired what the author meant by "less effectual," as the requirements of the operation seemed hardly met by such a result. Dr. Griffiths (Swansea) had seen satisfactory results from the operation; but in one case it was followed by a sharp attack of orchitis, which he regarded as a serious matter, and he would like to know in how many cases Mr. Spanton had met with the same result. For himself, he would be more inclined, when a truss had failed to cure or give adequate support, to cut down upon the sac and tie the canal. Mr. Norton (London) inquired how many deaths there had been in the thirty-four. Mr. Spanton, in reply, stated that there had been no deaths. He had used the expression "more or less" because, in one or two cases the patients had still to wear a truss, which now, after the operation, was effectual, but had not been so before. He had seen orchitis in perhaps a third or fourth of the cases, but regarded that as a very simple matter.

### AFTER THIRTY-EIGHT YEARS' PRACTICE.

BY HENRY S. CHASE, M. D., D.D.S., ST. LOUIS, MO.

(Prepared by invitation of the Wisconsin State Dental Society, for their Eleventh Annual Meeting, in Milwaukee, July 19, 1881.)

After thirty-eight years' practice in the profession of dentistry, I have seen many changes and some improvements. I hope and think my own experience and close observation of the results produced by my brother dentists have been of benefit to my patients. And now I will tell you what my practice is to day.

Fill all cavities not in sight with the best amalgam to be had.

Test every new batch of alloy by glass tubes and aniline alcohol.

Varnish all cavities before filling with anything; use sandarac or shellac for gold or amalgam cavities; copal ether for all bone fillings.

Fill front teeth and cavities in sight with phosphate of zinc, and paint over the surface of the same fillings every eighteen months with the same substance.

Protect *cervical* margins of cavities filled with metallic filling with gutta-percha.

Have clear separations between filled teeth, so that cleanliness may be insured.

Non-cohesive gold foil, in cylinders, preferred to any other form.

After killing pulps, wait ten days, in order that the dead part may separate with the living part, before removing the dead part. After removal, cleanse root-canals with alcohol, and fill with thick solution of sandarac or shellac. In treating periostitis from dead pulp, drill needle-sized hole through to the pulp-chamber, exactly at the edge of the gum and beneath it.

Fistulæ to be cauterized through roots with *creasote*, until it appears on the surface of the gum.

Chronic and constitutional periostitis, improperly called "Rigg's disease," to be treated constitutionally according to indications, and *locally* by chloride of zinc, carried under the gum as far as a small and thin instrument will go without cutting the tissues. In nearly all cases *salt* is prohibited as food, and all stimulating and fiery condiments. The juice of one lemon each day is to be swallowed. Once or twice a week the roots to be scraped and the zinc applied.

Be careful of *over*-treatment.

At the age of twelve years four permanent teeth to be extracted. Sometimes the four sixth-year molars, or four bicuspids, selecting the poorest of these teeth. This improves the condition of the oral cavity. The remaining teeth are better preserved, and the spaces sufficiently filled before twenty years of age.

Spiral springs to hold an under set of teeth in place are of great service. They are invaluable when the under jaw is very flat. They were much used thirty years ago, and should never have been so generally abandoned by the profession. (Look in the January, 1881, number of the *Dental Cosmos*, and see the article of Dr. Evans, of Washington, with illustrations.) In my own practice, I place the *pins* between the bicuspids, both above and below. Length of spiral two inches, of ten-carat gold—*never silver*.

Amalgam alloys were so unsatisfactory twenty years ago that I would not use them, except in rare instances. Six years ago there were one or two good ones for sale. To-day there are better ones than six years ago. We have a right to expect that far better filling materials will be used ten years from now than we are able to obtain at the present day. We especially need an improved "bone" filling.

I take pleasure in saying that I have made and am using an amalgam alloy which is perfectly *alcohol-tight.* Aniline alcohol is entirely kept out of glass tubes filled with this alloy. In Dr. Flagg's book on "Plastic Fillings" is hinted the use of zinc, and to that I am indebted for using zinc in the formula which proves to give such satisfactory results. My formula has not been published, and I take pleasure in giving the Wisconsin State Dental Society the first written formula. My new alloy is made as follows: Melt forty pennyweights of pure silver; add to this thirty pennyweights pure tin; stir it, then add five pennyweights of antimony and five pennyweights of pure tears of zinc. When mixed, add thirty pennyweights of pure tin again; stir, and throw on the surface of the "melt" one-half ounce of beeswax to burn off; and while burning, pour the "melt" into the *cup* of a vulcanizing flask to *cool.* Cut it up with very coarse file. Remove every particle of iron with horseshoe-magnet. This amalgam must be washed in alcohol while mixing with mercury. Squeeze it in dry buckskin. This amalgam is *whiter* for washing, and takes less mercury. Squeezing injures some amalgams; it does not hurt this. The amalgam pellets must be dry when placed in the cavity. This amalgam remains very white in the mouth. If *all* the tin should be melted at once, the antimony and zinc would never melt. If the antimony and zinc are put in the melted silver before the tin, then the antimony and zinc will burn up or oxidize. Varnishes seal up the ends of tubuli. The *gum* in the varnish is a poor conductor of heat and electricity. Gold fillings, being generally *leaky*, are improved by varnishing with sandarac alcohol, before they get wet with saliva or water. The varnish seals up the *leaks*, and becomes semi-hard by after-contact with the saliva. Leaky amalgam fillings are much improved by the same operation. —*Dental Cosmos.*

### A NEW DENTAL DISEASE.

A child, aged ten, whose teeth six months ago appeared to be all perfectly sound, came to me with toothache in the right lower canine.

I found that a large portion of the enamel had disappeared from the front surface of the tooth, as if it had been chipped violently off; the dentine was all exposed, but there was no softening or appearance of decay. The disease, which has commenced in several of the other incisor teeth, appears first as a small white spot in about the thickest part of the front surface of the enamel, which it seems to penetrate, and then, suddenly disintegrating, this comes away, and exposes the remaining sensitive enamel and the dentine. This disease is altogether a different thing from the gradual decay, or wear at the neck of the teeth, frequently met with in adults, for in this case the patient is only ten, and, as far as I have been able to ascertain, the incisors and canines never have been known to decay in the manner above described. We are often at our wits' end to cope with the increasing prevalence of caries in the teeth of the very young; and if this be (as I fear it is) a new form of destructive energy, the sooner it is recognized the better.—*N. Stevenson, M.R.C.S., in British Medical Journal.*

### CHRYSOPHANIC ACID IN PSORIASIS.

Chrysophanic acid has been used successfully for some time as a remedy for psoriasis. It is perhaps the best remedy we possess for that affection. Where, however, the skin affection is extensive, or the remedy too strong, it sometimes causes sickness and vomiting. It may be applied in combination with melted lard, or what is better, with vaseline, in the proportion of from 30 to 60 grains to the ounce. Dr. M. Charteris of England, has been using the remedy, in combination with vaseline, with complete success in quite a number of cases. His article is published in the *Lancet* for July, 1881. In a case where the disease (*psoriasis*) extended over the whole body the usual formula of 1 to 8 of vaseline was found too strong; nausea and vomiting occurred so that he was compelled to apply it of a much weaker strength, viz: 1 to 16. During his experience he learned one singular fact, that where the disease was nearly equal on both sides, or was symmetrical, the application of chrysophanic acid and vaseline to one side of the body acted equally on both sides. He took patients, so afflicted, covered the arm and leg with close-fitting flannel, so that nothing could touch it, and made the application to the arm and leg of the opposite side. The covered limbs recovered from the affection nearly, if not altogether, as soon as those receiving the ointment.

Cases affected for months and years, and which had resisted all kinds of treatment, readily yielded to this plan in from 10 to 14 days.

It would appear from the disappearance of the affection on one side by the application of the remedy to the other, and also from the sickness it occasioned, that the acid is absorbed into the blood and acts as a constitutional as well as local remedy. This fact explains the observations of Dr. Crocker, who applied the acid to one side of the body and turpentine to the other, and found the respective sides healed in about the same time. He concluded therefore, that turpentine was as good a remedy for psoriasis as chrysophanic acid.

From the above experiments of Prof. Charteris, it is evident that the acid acts both locally and constitutionally, and that in Crocker's case the disease yielded to the constitutional effect of the acid, and not to the turpentine.—*Pittsburgh Medical Journal.*

### ECZEMA OF THE NIPPLE.

Mr. George Lawson exhibited a breast which he had removed on account of long standing eczema of the nipple. In the reports of the Society for last year, after recording a case in which cancer had followed an intractable eczema of the nipple, he suggested that in cases of eczema of the nipple which had resisted for a length of time all treatment, "the breast should be removed, in anticipation of a disease which does not then exist." The case which he had now to relate was that of a lady who suffered from eczema of the nipple, which had resisted all treatment for over twelve months, and was extending. Feeling that the usual termination of such cases was diffused cancer, Mr. Lawson advised and accordingly removed the breast. The eczema presented the usual appearance of such cases. It was a red, scaly patch, raised slightly above the level of the skin, and extending for about two inches around the nipple. The breast was afterwards examined by Dr. Thin, and he discovered cancer-elements diffused throughout the organ. The preparation was now in the College of

Surgeons. The President referred to the serious difficulty surrounding such cases. It would be dangerous to assume cancer in every case of eczema of the nipple, and yet the latter often preceded the grave affection. Some time ago he had been called to examine a patient with eczema of the nipple, and contented himself with ordering the application of ointments only. He had learned at a later period that this patient was suffering from cancer of the breast. Mr. Lawson advocated excision of the whole gland, when eczema of the nipple had resisted all treatment.—*Lancet.*

### AMYL NITRITE AS A CARDIAC STIMULANT.

Dr. Edward T. Reichert, Newark, N. J., contributes to *The New York Medical Journal and Obstetrical Review* for July, 1881, an article in which, from a critical consideration of the most important of the literature bearing upon the subject, as well as from experimental data of his own, he argues that nitrite of amyl acts as a direct stimulant upon the heart. The author admits that the increased action of the heart under the influence of the drug may be due, to a certain extent, to its depressant effect upon the pneumogastrics, as shown by Filenhe, Mayer and Friedrich, Dugan, and Brunton ; but he thinks that the deductions of the first three of these observers must be accepted with allowance, because of the very indirect way in which they sought to decide this action. Dugan, whose view closely coincides with Pick's (that there is a compensatory relation between the action of the heart and the condition of the vaso-motor system, so that when the vascular channels are open the heart will naturally beat faster to overcome the excessive drainage, and *vice versa*), was misled by not discriminating between the action of the drug on the vaso-motor system and its effect on the heart. With the action of the vaso-motor system practically abolished (as was accomplished in Brunton's experiments, in three of which he compressed the aorta below the diaphragm and divided the spinal cord, the disturbing influence of respiration and struggles on the animal's part being done away with by means of curare), any change in the arterial tension must be the result of direct cardiac action ; and in all of Brunton's experiments there was a primary and marked rise of pressure, which equaled as much as a fifth of the normal. The nitrites affect the blood pressure in two ways—by stimulating the heart directly, and by depressing the vaso-motor system, especially the centers. They are direct stimulants of the heart, increasing the frequency of its action and the amount of work done in a given time. Clinical evidence supports this view ; for, were it otherwise, the action of amyl in chloroform poisoning, in collapse and syncope, and in heart disease accompanied by paroxysms of distress due to the weakening of its action, would either be *nil*, or else it would even aggravate the symptoms.

### EARACHE.

" In the course of practice you will often be called upon to attend a case of Earache. This means, pathologically speaking, acute inflammation of the membrana tympani. Now, in such cases you may quickly subdue the inflammation, relieve the patient from the excruciating pain he is suffering, and save him perhaps, from subsequent confirmed deafness. The treatment, from which such a very desirable result may be obtained, is similar to that which you will find so beneficial in analogous cases of eye diseases—viz: leeches behind the ear, hydrag. c. creta and belladonna powders, with warm fomentations."—*Wharton Jones* in *London Lancet.*

### EARACHE.

In the American Medical Association, Dr. Jacoby remarked that closing the mouths of infants and children, and simply blowing into the nose, is often a very valuable method of relieving severe earache ; and that, in a number of cases, he had obtained most excellent results from this procedure, the cause of the trouble probably being a catarrhal affection of the eustachian tube.—*N. Y. Med. Times.*

### BLOOD ENEMATA.

Dr. Sansom thus writes of the employment of blood in nourishing enemata: Ox blood is usually employed, but sheep's blood may be used. It is necessary that it be defibrinated the moment it is drawn Butchers understand this process, and will supply what is called " whipped" or " stirred " blood. It is, of course, requisite that the blood be fresh—that it be not kept more than a single day. In urgent cases, where there is no stomach digestion, two or three ounces of blood may be injected into the rectum every two or three hours; the fluid may be warmed by placing the containing vessel in hot water, but it is often borne equally well when cold. For chronic cases in which it supplements stomach alimentation, it is administered in quantities of from two to six ounces twice a day. In some cases it tends to promote constipation; in a very small percentage, the opposite condition of irritability.—*The Lancet.*

### THE ACTION AND USES OF ANTIPYRETIC MEDICINES ADMINISTERED INTERNALLY UPON SEPTICEMIA AND ALLIED CONDITIONS.

Prof. Binz read a paper on this subject at the International Medical Congress, of which the following is an abstract:

1. In the present state of our knowledge there are two modes in which antipyretic remedies may be conceived to operate; first, by increasing the discharge of the pyrexial heat; secondly, by checking its production.

2. The quantity of heat discharged may be augmented by direct withdrawal (tepid water), or by facilitating the circulation through the skin (digitalis, cutaneous irritants).

3. The production of heat may be lessened by repeated cooling of the surface, and especially by the internal use of antizymotics.

4. Febrile diseases commonly owe their origin to the introduction and rapid development of substances akin to ferments. Several of these have been shown to resemble yeast in being low vegetable organisms or derived from such organisms. They enter the glands, where they undergo multiplication, increase the metabolic processes, generate products of decomposition which exert a paralyzing action on the nervous system, and raise the standard of temperature throughout the body.

5. Owing to impaired action of the heart in certain stages of the disorder, or to contraction of the cutaneous vessels, the skin becomes anemic and gives off less heat than usual. The internal temperature rises accordingly.

6. Quinine, our chief antipyretic, acts by directly combating the efficient cause of the disorder, and by checking the abnormal metabolism going on in the body. The nervous system takes no part or only a secondary part in this operation. In intermittent fevers quinine prevents the paroxysms by attacking their infective cause. The paroxysms are not the essence—the substantive element—of the disease ; they are only a symptom of it. The substantive element is the poison deposited in the colorless corpuscles of many organs, especially the spleen. There are fevers without paroxysms and paroxysms without fever. It is just those intermittent fevers which run their course without paroxysms that are the most malignant. The malarial poison rapidly causes disintegration of the tissues and the blood, and so paralyzes the nerve-centers.

7. The reduction of acute splenic tumors by quinine depends upon the adverse influence exerted by the alkaloid on the infective poison to which the morbid over-action of the spleen and its consequent enlargement are due. "*Cessante causâ cessat effectus.*" Even a healthy spleen may be reduced in size by large doses of quinine ; the alkaloid vigorously checking the oxidation of its principal elements, the colorless corpuscles. Quinine has no direct influence on the vaso-motor nerves.

8. Quinine attacks the malarial poison with especial energy ; on this fact depends the so-called specific action of quinine in intermittent fevers. The same relation, but in a minor degree, subsists between quinine and the infective poison of enteric fever, between mercury and iodine and the poison of syphilis, between salicylic acid and the "irritant" in acute articular rheumatism.

9. An antipyretic which in one disease instantaneously arrests the fever may be wholly powerless in another. The difference depends on the fact that the various antizymotics act very unequally upon the individual *schizomycetes* and ferments ; one will paralyze them rapidly, by another they will hardly be affected.

10. The past history of therapeutics and recent achievements in the domain of etiology and pharmacology entitle us to assume that by persistent scientific inquiry and practical observation we may succeed in discovering a specific antidote for every species of infective or septicemic malady.—*British Medical Journal.*

### MOUTH WASH FOR TOBACCO CONSUMERS.

---

C. Graham, M. D. Chicago, writes: Bromo-chloralum, twenty to thirty drops, in a tablespoonful of water, forms an excellent deodorizing mouth wash, where it becomes desirable to at once destroy the effect upon the breath of tobacco smoking or chewing. It acts like a charm—it being odorless itself, yet destroying instantly the after-effect of the weed upon the breath.—*Amer. Specialist.*

### THE HIPPOCRATIC OATH.

The most curious medical monument of antiquity is the famous Hippocratic oath, the faithful observance of which secured good success in life, and general esteem. The oath is as follows :

" I swear, by Apollo, the physician, by Æsculapius, by Hygeia and Panacea, and all the gods and goddesses, calling them to witness that I will fulfil religiously, according to the best of my power and judgment, the solemn promise and the written bond which I now do make. I will honor, as my parents, the master who has taught me this art, and endeavor to minister to all his necessities. I will consider his children as my own brothers, and will teach them my profession, should they express a desire to follow it, without remuneration or written bond. I will admit to my lessons, my discourses, and all my other methods of teaching, my own sons, and those of my tutor, and those who have been inscribed as pupils and have taken the medical oath ; but no one else. I will prescribe such a course of regimen as may be best suited to the condition of my patients, according to the best of my power and judgment, seek to preserve them from anything that might prove injurious. No inducement shall ever lead me to administer poison, nor will I ever be the author of such advice : neither will I contribute to an abortion. I will maintain religiously the purity and integrity, both of my conduct and my art. I will not cut any one for the stone, but will leave that operation to those who cultivate it. Into whatever dwellings I may go, I will enter them with the sole view of succoring the sick, abstaining from all injurious views and corruption, especially from any immodest action towards women and men, freeman or slaves. If during my attendance, or even unprofessionally, in common life, I happen to see or hear of any circumstances which should not be revealed, I will consider them a profound secret, and observe on the subject a religious silence. May I, if I rigidly observe this, my oath, and do not break it, enjoy good success in life and in (the practice of) my art, and obtain general esteem forever. Should I transgress and become a perjurer, may remorse be my lot."—*Cincinnati Lancet and Clinic.*

### SALICYLIC ACID FOR COLD IN THE HEAD.

Dr. H. H. Fair says : I have been using salicylic acid for some two years for the frontal pain with coryza and increased lachrymation, occasioned by cold. This affection sometimes gives great pain, and is often very difficult to reach with the usual remedies employed. Salicylic acid given in ten grain doses every two or three hours will give prompt relief. In my experience the third dose has hardly ever been necessary.—*Therapeutic Gazette.*

### PHOSPHIDE OF ZINC IN LOCOMOTOR ATAXIA.

Dr. Hastings Burroughs (*Medical Press and Circular*) gives this drug in one-eighth grain pills—one a day for a week, and then two daily, and so on up to five. He has treated his cases successfully thus far.—*Phil. Med. Times.*

NITRITE OF AMYL will be found a very effectual remedy in chordee and painful priapism. We have recently prescribed it for those conditions with very satisfactory results. Three to five drops by inhalation is the proper dose.—*St. Louis Clin. Review*, June, 1881.

### CANCER OF THE BREAST.

M. Després (*Le Prog. Med.*) recalled a case where the cancer reappeared nine years after removal, coming outside the cicatrix. M. Delens cited a case where the interval was five years. M. Le Fort did not endorse the opinion of M. Desprès, that suppuration was necessary in order to prevent a relapse. He thought that the duration of an inflammation, which a long suppuration necessitated, could only favor the return of certain forms of cancer, as,for example, epithelioma. Reunion by first intention was certainly to be preferred whenever possible. M. Lucas Championnière thought that Listerism, which sought for and obtained union by first intention,had shown that relapses were not more frequent or premature when suppuration was avoided. M. Gillette thought the rule which fixed one year as the return epoch was too absolute. He knew of several cases where the interval was much greater. One case, among others, that of an open cancer in a diabetic patient, was cured in three weeks; no symptom of return at the end of two years. (T. M. S.—*N. Y. Med. Times.*

### ACONITE IN REMITTENT FEVER.

Dr. G. Bamford (Practitioner) claims the following results for this drug in remittent fever: 1st, it reduces the temperature; 2d, it reduces the pulse rate; 3d, it cleans the tongue and restores the digestive function; 4th, it produces sleep; 5th, it increases the quantity of the urine; 6th, it promotes perspiration.—*Ks. Med. Index.*

### OXALATE OF CERIUM IN PERTUSSIS.

Dr. Morje, in accordance with Dr. Clarke's recommendation, has tried oxalate of cerium in the spasmodic stage of whooping-cough. Not only was the frequency of the attacks reduced, but their intensity was also lessened, in each case giving the patient a good night's rest; and invariably shortening the second and most severe stage of the disease. The remedy was employed in ten cases, of which seven were females. Two of the cases were complicated with other diseases. The mode in which the oxalate of cerium was administered was always the same, a single dose each day before breakfast. The ages of the patients under observation ranged from one to seven years, and the oxalate was administered in half-grain to three-grain doses. In every case the remedy was continued one week longer than there was any existence of the whoop, to obviate the possibility of a relapse. The advantages claimed for oxalate of cerium are that it decreases the attacks, and thereby reduces the violence of the disease, often checking it instantly. It is easily administered, as only one dose is required in 24 hours. Nocturnal quietude is insured. The possibility of complications is lessened.—*Ex. Gailard's Medical Journal.*

### OPERATION UPON THE RADIAL NERVE BY SUTURES.

A laborer, æt. 31, received, in consequence of the falling of a ceiling, several injuries, among which was a severe bruise upon the outside of the right arm, just below the middle of the humerus; simultaneously there appeared paralysis of the extensors of the forearm and hand. At the site of the bruise the skin sloughed and an abscess formed, which was incised. Paralysis persisting after healing of the part, Langenbeck decided that the radial nerve was divided, and two and a half months after the injury decided to perform an operation. The ends of the nerve were found separated to the extent of two centimeters (three-fourths of an inch); they were freed from attachment, freshened and brought together by a cat-gut suture that was passed through the nerves themselves; this union was effected only with considerable traction. The exsected cicatrical tissue contained no nervous elements whatever. Healing took place without suppuration and after nineteen days contraction of the extensors could be excited by the Faradic current. One and a half months after the operation the patient could make considerable movement with the extensors.—*Berlin Klin. Woch.*

### COLLAPSE IN STRANGULATED HERNIA.

M. Verneuil (*Le Prog. Med.*) seeing the fatality which followed the operation of colotomy in those who were cyanosed and algid had ascribed it to pulmonary congestion. He operated lately on a case of strangulated hernia in this condition, with the result of the death of the patient 34 hours after the operation, with all the symptoms of a very intense pulmonary congestion. The autopsy revealed a double and complete pulmonary congestion of remarkable intensity. The kidneys presented all the signs, in a marked degree, of the third stage of Bright's disease. The lowered temperature, anuria, and the post-mortem developments pointed to uræmia as the cause of death. In this case then the symptoms are to be explained by a renal lesion. M. Despres thought that the obstruction of the intestines by waste matters caused distention, and finally pulmonary congestion and death. He was in doubt whether Opium—first advised by the English physicians—was not contra-indicated in these cases. Manec, who did not use it, but purged his patients, had had excellent results.

M. Trelat said that his rule had been, nourishment in small quantities and Opium. As a result of his experience in 450 cases of colotomy, he concludes that delayed operations are dangerous, but that early ones are successful.

M. Lefort claimed priority in the employment of Opium after these operations. He still thought Opium the best remedy against these accidents of abdominal stupor due perhaps to the irritation of the splanchnic nerves. M. See did not think it was necessary to re-establish the course of the intestinal waste during the first few days, and called attention to the serious results following the sudden re-establisment by nature itself.

M. Verneuil said all the patients in whom intestinal action was not re-established within the first days did not die, and hence some other cause was necessary. He had also found in the liquid of the sac a large quantity of bacteria, and hence the washing of the sac with a strong solution of phenic acid was advisable.—*N. Y. Med. Times.*

### INFLUENCE UPON THE EYE PRODUCED BY SECTION OF THE TRIGEMINUS.

M. Poncet (*Le Prog. Med.*) in examining the eyes of rabbits, after section of the trigeminus, at intervals of 8, 15, 30 days, and a year has observed the following facts : 1, in the nerves of the cornea, whose degeneration has been so well described by Ranvier, he found, at the end of a year, a complete regeneration of the corneal plexus, in a manner absolutely different from the normal course. In the midst of these nerve excavations, but incapable of explanation, we find nerve sheaths, in which the old tubuli have not been regenerated; 2, the keratitis, which may be accompanied by an exudation into the anterior chamber, is seated in the superficial layers of the cornea. Iritis, suppuration of the process, posterior choroiditis, trouble of the humors, migration of pigment into the retina, and exfoliation of this membrane do not exist ; but, in the retina the deepest layers are the seat of an œdema, characterized either by the presence between the optic fibers of œdematous masses, or by the hypertrophic degeneration of the ganglionic cells, or by the increase in volume of the protoplasm of the internal sheaths. The remaining layers are healthy. These alterations differ essentially from those produced by the section of the optico-ciliary nerve.

### SMALLPOX IN A FŒTUS IN UTERO WHEN MOTHER HAD BEEN VACCINATED AND NEVER HAD VARIOLA.

Prof. Labbe says that at the birth of the child the pustules seemed to have existed seven or eight days ; they were larger than the ordinary, umbilicated and differed from pemphigus and other skin affections. The child died a few moments after birth.

Vinal explained the case in the following way : From the condition of the child's skin and the history it must be assumed that at the time of the conception, in November or December, 1879, the father of the child must have had variola. The mother of the child had been vaccinated in early life and hence had not taken the disease from the man, nor had she been afflicted since. It must follow, therefore, that the poison was in the semen, and remained latent in the fœtus till shortly before birth.—*Medizinische Neuigkeiten.*

### THE POISON OF TYPHOID.

Prof. Klebs announces in the *Archiv. fuer Experimentelle Pathologie* the discovery of the specific poison of typhoid fever. It is, according to him, a bacterium, rod-shaped, and about .004 inch long. It is found generally diffused in those organs which are most affected by the disease, and has been observed only in connection with typhoid. It does not appear that experiments were made by inoculation, without which the discovery can hardly be announced as such.

### TREATMENT OF SPRAINS BY COLLODION.

Dr. A. N. Blodgett, in the *Boston Med. and Surg. Jour.*, p. 294, 1881, relates that in the winter of 1878 he sprained his own ankle, and having tried the usual methods of treatment with very indifferent success, was resigning himself to let the sprain take care of itself, when it occurred to him that the application of Collodion, so prepaed that it would contract in drying, might be of some service. He made the trial and was surprised and pleased at the result. For a few minutes no appreciable effect seemed to follow, but after several coatings there commenced a contraction of the whole layer of Collodion from all directions at once, to a much greater degree and in a much more efficient manner than any bandage could possibly effect. As the Collodion films cracked and divided into scales, these were picked off and fresh coatings applied in succession, until, in the short space of three days, the ankle was restored to its original size, and there was a total absence of pain and tenderness in the joint. In a week he found himself quite well, and has never had a relapse.

Dr. Blodgett cites eight cases successfully treated by Collodion. Among the advantages of this mode of treatment are, briefly, prolonged elastic compression in parts notoriously difficult to bandage properly ; waterproof protection to the skin from external irritants or application ; hermetical sealing up of wounds in the region of the strain or sprain ; constant access to the part without the removal of dressings ; an uninterrupted view of every part of the injured limb ; reduction of heat in the tissues ; great acceleration of the process of healing with perfect restoration of function ; a great degree of immunity from relapse ; and absolute simplicity in application.

" So far as my limited experience warrants an opinion of Collodion in the treatment of strains and sprains, I am inclined to consider it by far the best, simplest, and most satisfactory method I have ever known. The degree of contraction depends much upon the quality of Collodion employed. The so-called contractile Collodion must be used for this purpose. To obtain the contractile effect of Collodion it is necessary to apply several coats successively, one upon the other. I think I have never applied less than six layers, which is easily accomplished, as the Collodion dries very quickly, and a second coat can be applied almost as soon as the first is finished."

### SYPHILITIC ALTERATIONS OF THE TEETH.

Mr. Parrot has devoted several lectures to the description of these alterations, described by some authors as being of convulsive origin, and by others as of a rachitic nature, whilst the author regards them as one of the products of hereditary syphilis. The following is a brief outline of the argument used by him to enforce his position: The typical alteration is found in the first molar, where it is constant if existing in other parts of the mouth. The altered part seems free in the middle of the teeth, and appears on a lower level than the sound part, from which it is separated by a sort of furrow. It is yellow, of an ochry aspect; the cuspids are more pointed and filled with small elevations like grains of sand, and extremely friable. On the other hand, the part of the tooth upon which these altered parts rest is normal, often covered as if with a coat of enamel. The peculiarity of this alteration is the sort of retraction which the tooth has undergone at the same time, and which has given rise to the separation between the healthy and affected parts.

The cup-shaped atrophy, next described, may present itself alone or associated with other varieties. It is more particularly observed upon the upper middle incisors. The tooth is generally large and high, and on the anterior and posterior face are observed small depressions of varying numbers—from one to eight. Their diameter does not exceed one millimetre, and they are in a horizontal row, united by small grooves or separated by a fold of enamel. At their level the tooth is not enameled; it is of a dirty yellow, and the dentine is often exposed. This form of atrophy is very remarkaable, and seems to be the elementary form of other varieties.

The third form is the sulciform atrophy. It is rarely found in the molars; it occurs chiefly in the incisors, and consists in horizontal furrows in numbers from one to three—rarely four—always horizontal and parallel near the maxillary border. A fourth variety is a hatchet-shaped atrophy. This is observed in the first dentition, only in the incisors, and almost always in the superior middle. This alteration is not primitive, being produced after the complete development of the tooth, whilst the other forms described commence when the tooth is still in its sac. The fifth variety is that to which Hutchinson has called attention, consisting of notches, occurring chiefly in the superior middle incisors, but being also found in others.

All of these varieties may be more or less considerably modified, but are almost always accompanied by certain consecutive alterations. First of all is the color, which may be modified, even in very careful persons, becoming yellowish or even greenish. In those exposed to different dusts, these attach themselves to the surface of the teeth, and give rise to various colors. There is also an abundant deposit of tartar. By far the most important consequent alteration is caries. The molars are lost early, the jaw is atrophied, and spaces, as normally seen in horses, are soon established.

There are, however, changes in the teeth which must not be confounded with those described. Thus, at the second dentition, the incisors often present at their free edges saw-like notches, then it may occur that from the friction the incisors may cut a bevel-edge on each other. The hatchet-shape may be determined by caries. And, again, there exist normally in some subjects true furrows. It is therefore necessary to be careful in making a diagnosis.—*St. Louis Medical and Surgical Journal.*

### HOT WATER FOR SWEATY FEET.

Dr. Gay, taking a hint from what he witnessed during a recent sojourn at the Hot Springs in Arkansas, has cured sweaty feet in many instances by simply directing the feet to be soaked for hours every day in water as hot as can be borne.—*Ohio Med. Reporter.*

### SYPHILITIC TUMORS OF THE BONE IN INFANCY.

M. Després (*Le Prog. Med.*), in reply to M. Lannelongue's communication on the above subject, reports a case of a young man, 19 years of age, who had a diffuse tumor of the right tibia, with lengthening of the leg of 3 cms., another tumor, but more limited, of the left tibia, a suppurative osteitis (gumma) of the malar region, which had been preceded by a frontal exostisis, now disappeared. This young man had never had syphilis, and the most minute enquiries failed to detect any syphilitic accidents in the parents. But as confirming, in some degree, the theory advanced by M. D., of the relationship between osseous tumors of infancy, and tuberculosis in the parents, one of the uncles of the patient had died, at the age of 32 years, of albuminuria and pulmonary complications.

M. Lannelongue remarked the resemblance between this case and those presented by him, and thought the trouble was of syphilitic origin, especially as there was on the palatine arch the cicatrix of a former ulceration. He thought an anti-syphilitic treatment would remove the disease.

M. Trelat, on the other hand, thought the case was one of chronic osteomyelitis, incident to youth. The marked lengthening of the bone, very rare in syphilis, was in favor of the above diagnosis. The palatine cicatrix might be the result of an abscess, or a light attack of gangrene, occurring in infancy. (T. M. S.)

### COUPLAND ON ANÆMIA.

Dr. Sidney Coupland, at the conclusion of his Gulstonian lectures on anæmia, gives a general review of the treatment (*Brit. Med. Journ.*, April 1881, p. 634). The necessity of proper diet and general hygienic measures are self-evident. Of all medicinal agents, iron maintains the pre-eminence. How it acts there is no satisfactory evidence to show; but, as Trousseau remarked, iron in chlorosis is, in heroic doses, as much a specific as mercury in syphilis; and our recent methods of calculating the red corpuscles enables us to accurately estimate the rapid action of iron upon the constituents of the blood. The sulphate, in large doses, is well borne in almost every case of pure chlorotic anæmia. Next to iron, and in some cases preferable, stands arsenic. Phosphorus is useful in some cases. Manganese has disappointed many. The subject of transfusion of blood is one that demands the most thorough examination; of its value in cases of anæmia due to sudden loss of blood, there is no question; but in cases of pernicious anæmia, its value is not yet decided. In twenty such cases in which it has been carried out, six recovered, one not permanently; and all the successful cases occurred in Dr. Quincke's practice.—*Lond. Med. Rec.*

### TREATMENT OF PUERPERAL CONVULSIONS BY HYPODERMIC INJECTION OF MORPHIA.

S. Maberly-Smith, resident surgeon at the Lying-in Hospital, Melbourne, Victoria, having found the usual treatment for puerperal convulsions, by chloral, bromide of potassium, bleeding, and chloroform, very unsatisfactory, tried hypodermic injection of morphia. From fifteen cases treated in this manner he has come to the following conclusions:

The quantity of morphia to be injected is from one-fourth to one-third of a grain, according to the severity of the case. The simple solution of morphia is more efficacious than morphia and atropia combined ; one large dose is better than two smaller ones.

Patients suffering from puerperal eclampsia, whether sensible or insensible, appear to resist the dangerous effects of the drug ; it seems to have no bad consequences in cases in which, under ordinary circumstances, morphia would be strongly contra-indicated. It has been injected in women who were insensible with stertorous breathing, congested lungs and faces, and contracted pupils, in every case with the best result.

After injection the patient may have one fit before the drug has had time to act, but never has another for hours, provided that the injection is proportionate to the severity of the attack. If at the end of some hours the patient has another fit it is generally a slight one, and a smaller injection than the first should be given. No case of puerperal eclampsia has died in the Melbourne Lying-in-Hospital since this treatment has been adopted.

### CHEMICAL ASPECT OF SUDDEN DEATH BY CHLOROFORM.

Dr. Latham, in the *Lancet*, February, 1881, p. 216, explains, chemically, why death occurs so suddenly in some cases under the influence of chloroform. Hoffman showed that, by means of chloroform, the amides of the vital fluids are converted into isocyanides. If, then, in a person whose co-ordinating chemical centre is in feeble state, blood, charged with chloroform, pass from the lungs to the heart, and at once, though the coronary arteries come into contact with the muscular tissue, one constituent of the muscle will be decomposed, and the transformed tissue will no longer respond to the nervous stimulus, but become a mere bag, into the right side of which the blood, from the pressure in the veins, will flow, and the patient will die with distended heart.

### ARTIFICIAL IMPREGNATION.

In a recent communication to the Société de Biologie, Dr. De Sinéty discussed the subject of sterility in the male. He was inclined to think that in families without children the cause was oftener in the male than had generally been supposed. The usual test of procreative power on the part of the male is an examination of the spermatic fluid. If spermatozoa are found, it is considered evidence that the man is potent. In three instances described by Dr. Sinéty, however, this conclusion seems to be made doubtful. In these cases there seemed to be nothing in the condition of the wives to explain their sterility. Spermatozoa were found in the spermatic fluid of the husbands. In order to be sure that the spermatic fluid reached the interior of the uterus, artificial impregnation was resorted to. Shortly after the menstrual flow this was repeated, at intervals, six times, but without avail. The fresh spermatic fluid had been examined, and found to contain many spermatozoa; but it was at last noticed that these spermatozoa, though very numerous, were for the most part immobile, and that movements soon ceased even in the few where they were first noticed. The observer's attention was awakened by this, and he examined the fluid in the two other cases, with the result of finding the same condition. Two of the patients had tuberculosis, and the third was much debilitated.—*N. Y. Med. Times.*

### APOMORPHIA IN DISEASES OF CHILDREN.

Impressed by the favorable reports published by Kormann with respect to the expectorant properties of apomorphia, Kushel tried this drug in seventeen cases of bronchitis and catarrhal pneumonia, in patients ranging from one-half up to ten years of age. There were eleven cases of bronchitis complicating measles, with high temperature (up to 41 deg. C.=105.8 deg. F.); one case of variola, with intense bronchial catarrh; two cases of catarrhal pneumonia; and, finally, three cases of obstinate bronchial catarrh with very tenacious sputa. In the last-mentioned cases muriate of ammonia and ipecac had previously been administered without loosening the cough ; in the remaining cases apomorphin was given from the first. In a short time, usually within the first twenty-four hours, previously dry rales gave place to moist ones, expectoration was facilitated, and when fever was present the temperature sank. All of the patients got well. The remedy caused no vomiting and no interference with digestion. The method of administration and dosage was that recommended by Kormann (*Jahrbuch für Kinderheilkunde*, *N. F.*, vol. xv., p. 180). For an infant one year old, the single dose should not exceed 0.001 (1-60 gr.) every two hours, *i.e.*, about ten times a day; for every additional year up to eleven years a half-milligramme is to be added to the individual dose; after eleven years one milligramme. Thus:

| | |
|---|---|
| ℞ Apomorphin, hydrochloric. crystall, . . | 0.02 |
| Acid. hydrochloric. dil., . . . . | gtt. iij |
| Syrup. sacchari s. senegæ s. ipecac, . . | 20.0 |
| Aq. distill., . . . . . . | 30.0 |

M. D. in vitro cœruleo. S.—A teaspoonful every one to two hours, for a child of three years.—*Med.-chirurg. Rundschau*, April, 1881.

### RARE CASE OF SELF-MUTILATION.

Dr. Thiersch related the case of a man who had circumcised himself at the age of eighteen. In 1870, being then married and a father, he slit up the hypogastrium from the symphysis pubis to the umbilicus, so that the omentum protruded; his object being, as he said, to obtain a view of his interior. Although the knife which he used was dirty and blunt, the wound healed after the removal of the omentum. A year later, he laid open one side of the scrotum. The prolapsed testicle was replaced while he was in hospital, and the wound healed without interruption. In 1880, he again laid open his abdomen; the wound healed in fourteen days, notwithstanding prolapse of the omentum. In May, 1880, he removed his right testicle, and himself sewed up the wound. Four weeks later, he treated the left testicle in the same way; the spermatic cord, however, escaped, and a hæmatoma, as large as a child's head, was formed, on account of which he was admitted into hospital. The man acted under the influence of an uncontrollable impulse, and had no quiet until he was completely castrated.—*Medical Gazette*.

Conium in Bronchial Catarrh.—Succus conii is recommended by Dr. J. J. Barnes (*British Medical Journal*), in bronchial affections in which irritation of the tracheo-bronchial membrane after cold is the chief feature. The dose is 15 to 20 minims every four hours.

## Figures and Tables

**Fig. 1. f1:**
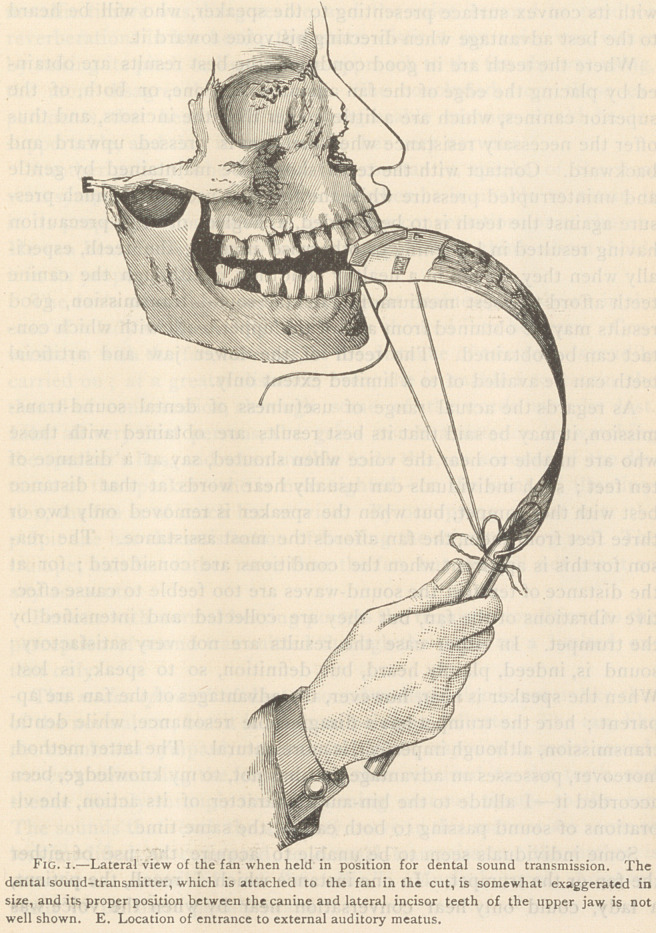


**Fig. 2. f2:**